# Translational Utility of Liquid Biopsies in Thyroid Cancer Management

**DOI:** 10.3390/cancers13143443

**Published:** 2021-07-09

**Authors:** Ayanthi A. Wijewardene, Marthe Chehade, Matti L. Gild, Roderick J. Clifton-Bligh, Martyn Bullock

**Affiliations:** 1 Department of Endocrinology, Royal North Shore Hospital, Sydney, NSW 2052, Australia; matti.gild@sydney.edu.au (M.L.G.); roderick.cliftonbligh@sydney.edu.au (R.J.C.-B.); martyn.bullock@sydney.edu.au (M.B.); 2 Faculty of Medicine and Health, University of Sydney, Sydney, NSW 2052, Australia; mche2952@uni.sydney.edu.au

**Keywords:** liquid biopsy, circulating tumor DNA (ctDNA), cell free DNA (cfDNA), microRNA (miRNA), thyroid cancer

## Abstract

**Simple Summary:**

Thyroid cancer remains a challenging malignancy, and it is difficultt to scale appropriate monitoring and therapy. Monitoring of the outcomes of thyroid cancer treatment currently relies on invasive tissue biopsies or repeat imaging involving radiation exposure. Liquid biopsies are a novel technique, which assess for common mutations associated with cancer through a simple blood sample. This review will assess the utility of liquid biopsies in the diagnosis and management of thyroid cancer and outline future directions which may optimise patient outcomes.

**Abstract:**

Liquid biopsies are a novel technique to assess for either circulating tumor cells (CTC) or circulating tumor DNA (ctDNA and microRNA (miRNA)) in peripheral blood samples of cancer patients. The diagnostic role of liquid biopsy in oncology has expanded in recent years, particularly in lung, colorectal and breast cancer. In thyroid cancer, the role of liquid biopsy in either diagnosis or prognosis is beginning to translate from the lab to the clinic. In this review, we describe the evolution of liquid biopsies in detecting CTC, ctDNA and miRNA in thyroid cancer patients, together with its limitations and future directions in clinical practice.

## 1. Introduction

Thyroid cancer is the most common endocrine malignancy, and its incidence is increasing, with approximately a quarter of million new cases diagnosed globally in 2017 [1,2]. The majority of thyroid cancer patients have an excellent outcome, with 85% 10-year survival [3]. Recurrence can occur from months to years from initial diagnosis. Nearly 50% of high-risk patients will have a recurrence within 5 years [4,5]. Early identification of patients with recurrence is crucial to ensure accurate assessment and less invasive treatment options. In current practice, surveillance for thyroid cancer recurrence currently relies on serum biomarkers (including thyroglobulin in differentiated thyroid cancer (DTC) and calcitonin in medullary thyroid cancer (MTC)), radiological screening and invasive biopsies. While these modalities are the gold standard of care, they each have limitations, creating a role for new monitoring techniques in thyroid cancer management.

Liquid biopsy is the sampling and analysis of blood or other bodily fluids for informative biomarkers, such as circulating cell-free-DNA (cfDNA), microRNAs (miRNAs), proteins and metabolites. Liquid biopsies have shown to be a powerful clinical tool for several cancers, including breast, bowel, and lung cancer. In recent years, there has been an increase in the literature assessing the role of liquid biopsies in thyroid cancer. Our review will describe the utility of liquid biopsies in detecting common mutations in thyroid cancer patients, together with limitations and future direction in clinical practice.

## 2. Modalities Used in Clinical Practice to Monitor Recurrence in Thyroid Cancer

### 2.1. Tumour Markers

Thyroglobulin (Tg) is the most abundant protein in the thyroid gland and is generated in thyroid follicular cells. The iodination of Tg is integral in thyroid hormone production [6]. While Tg is used universally to monitor biochemical recurrence in DTC patients, it has some limitations in clinical practice. Firstly, as thyroid cancer becomes poorly differentiated, cells no longer express Tg, making it a less reliable marker of recurrence [7]; up to 15% of patients with stimulated Tg < 1ug/L were found to have a distant metastasis, with lower Tg seen in tumors with an aggressive histology and *BRAF^V600E^* mutations [8,9]. Secondly, 30% of patients with DTC have anti-Tg antibodies, which interfere with the accuracy of the Tg assay [10]. Furthermore, the utility of Tg is diminished in the setting of residual normal thyroid tissue following hemithyroidectomy or subtotal thyroidectomy, used for low-risk disease [11].

Calcitonin (Ctn), is the gold-standard serum biomarker for monitoring MTC. It is a 32 amino acid peptide that results from the cleavage of procalcitonin, which are secreted from the C cells of the thyroid gland. Despite new assays for Ctn having high sensitivity, falsely elevated levels can occur in conditions such as sepsis, chronic renal failure and non-thyroidal malignancies; therefore, Ctn needs to be appropriately interpreted in the clinical setting [12]. Ctn levels can also vary with changes in physical activity, age and weight, making it difficult to monitor treatment response over time [13].

### 2.2. Imaging

Limitations also exist in the assessment of structural recurrence with current imaging techniques. Imaging modalities used to monitor recurrence include neck ultrasounds (US), iodine scans (I-131) computed tomography (CT) and positron emission tomography (PET) scans. Neck US are limited by their capacity to differentiate benign nodules from thyroid bed recurrence [14], while repeated CT and PET scans incur ongoing radiation exposure, thus increasing the risk of secondary malignancies [15]. Therefore, while crucial in the management of thyroid cancer, the available imaging modalities can be expensive, time-consuming and confer further radiation exposure when used routinely to monitor disease progression.

## 3. The Need for New Screening Tools in Thyroid Cancer: Identifying Molecular Markers

The identification of oncogenic drivers in DTC has led to greater understanding of its pathogenesis, and some of these are now routinely used in practice. Over 70% of DTCs harbor acquired somatic mutations in *RAS* (H, K, N), *BRAF*, *NTRK1*, or *RET* rearrangements [16]. These molecular markers are used to guide prognosis, treatment choices and screening for resistance mutations. Molecular-based therapies are now in clinical practice for refractory and aggressive thyroid cancer, with tyrosine kinase inhibitors (TKI), lenvatinib and sorafenib being shown to improve progression-free survival [17,18]. More specific kinase inhibitors target driver mutations, e.g., selpercatinib, in *RET*-mutated thyroid cancers [18,19,20]. TKIs are limited by drug toxicities including diarrhoea, palmar-plantar-erythroysesthesia and hypertension, and require close monitoring to maximise efficacy.

In current clinical practice, the identification of these important molecular markers is limited to screening tumoral tissue, which requires invasive biopsies. If tumoral samples have not been banked, then screening for these mutations may be not possible, hindering patients’ access to molecular-based therapies. Furthermore, as screenings of molecular markers are limited to invasive tissue biopsies, they cannot easily be used to identify new resistance mutations or monitor treatment response.

Liquid biopsies could comprise a novel technique to screen for these mutations via a simple peripheral blood test, opening the possibility for use in pre-operative assessment, treatment initiation and monitoring treatment response. The advantage of liquid biopsy is its ability to carry out non-invasive sampling for molecular profiling, which would normally warrant invasive biopsies with the risk of seeding, as well as capturing diseases that are not radiologically evident [21]. As it is relatively easy to perform, has a non-invasive nature, and allows for frequent sampling to track tumor evolution, liquid biopsy is an appealing option for both patients and clinicians.

## 4. Genesis of Liquid Biopsy

**Figure 1:**
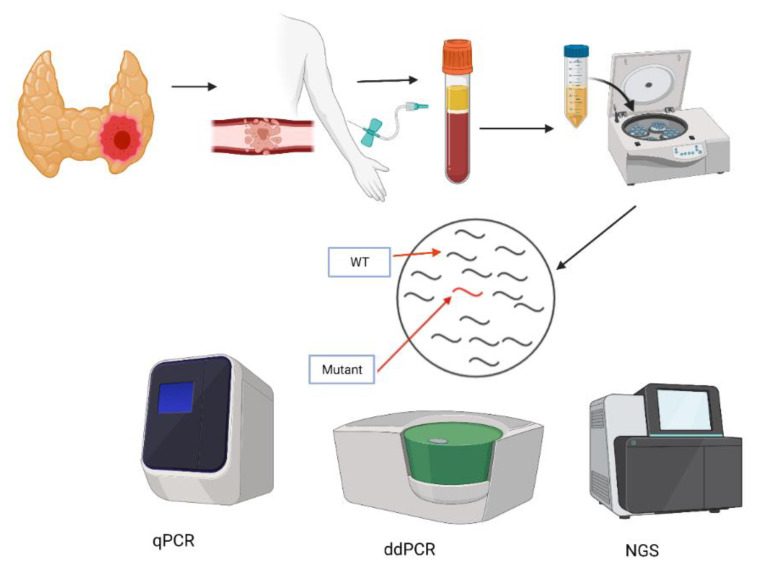
Detection techniques for liquid biopsies. (Created with BioRender.com, accessed on 2 March 2021.) Peripheral blood samples are collected and centrifuged to separate plasma from whole blood. DNA and RNA are extracted from plasma. Targeted mutation screening can be undertaken using Real-Time PCR (qPCR) and digital droplet PCR (ddPCR). Next-generation sequencing (NGS) allows for the screening for unknown mutations.

Liquid biopsy is the sampling and analysis of blood or other bodily fluids to assess circulating tumor cells, nucleic acids, proteins and metabolites (Figure 1). cfDNA was discovered in 1948 with the detection of free circulating DNA fragments in patients with systemic lupus erythematous [22]. cfDNA is thought to originate from the apoptosis of lymphoid and myeloid cells in normal cellular homeostasis [23,24]. Healthy older patients have higher levels of cfDNA than younger patients [25], which may reflect pathophysiological processes such as inflammation, diabetes, sepsis, and myocardial infarction [26,27]. Circulating tumor DNA (ctDNA) is, specifically, cfDNA released from apoptotic or necrotic tumor cells. Circulating tumoral cells (CTC) are metastatic tumor cells found in the circulation [28]. Both CTC and ctDNA have been used to monitor disease progression with cancer patients. ctDNA levels in cancer patients range from 0 to >1000 ng/mL compared to 0 to 100 ng/mL in healthy controls [24]. Liquid biopsies have also played a role in the screening of patients with a high risk of cancer, with 20% of those with CTCs obtaining a diagnosis of cancer within 12 months of testing [29].

Liquid biopsies are an effective tool in aiding decisions regarding oncology treatments. In non-small-cell lung cancer (NSCLC), *EGFR* mutation testing is used by clinicians to guide patients towards *EGFR* tyrosine kinase inhibitors [30,31]. ctDNA is effective in monitoring disease progression in metastatic breast cancer and colorectal cancers (CRC), with over 90% accuracy [32,33,34,35]. Liquid biopsies can also be effective in detecting new genetic mutations in CRC that can cause treatment resistance, which, when found early, can lead to the initiation of alternative therapies [36,37,38].

## 5. Methods of ctDNA Detection

Techniques for the genomic profiling of cancers by liquid biopsy can be divided into two main strategies. The first is a targeted approach, whereby tumor-specific biomarker(s), identified from a prior tumor biopsy, are assayed to monitor residual disease. PCR-based assays are ideal for rapidly interrogating one or a small number of mutation hotspots. Traditional real-time PCR with hydrolysis probes is the most rapid and cheap method of ctDNA detection, although its sensitivity in detecting mutations in background wild-type (WT) DNA is limited to a mutant allele frequency (MAF) of 10–20%. Fortunately, several different improvements to the original chemistry have led to drastic improvements in assay sensitivity. One of the most widely used of these newer techniques is competitive, allele-specific TaqMan PCR (castPCR) technology. Developed by Barbano et al., it achieves a sensitivity of 0.1% MAF by utilising a mutation-specific primer and a minor groove-binding blocker to suppress WT amplification [39]. Droplet-digital PCR (ddPCR) was developed to provide high precision and the absolute quantification of targets, and could potentially detect mutations at levels as low as 0.01%. MAF. The ddPCR assay is based on water–oil emulsion droplet technology that fractionates a DNA sample into thousands of droplets, which are then PCR-amplified using allele-specific hydrolysis probes. After the PCR, each droplet either contains mutant, WT or no DNA, thus allowing for an absolute estimation of the number of mutant copies and their concentration in the reaction under the assumption of Poisson distribution. Next-generation sequencing technologies (NGS)-based amplicon panels can simultaneously genotype many hotspots; however, as it is a time-consuming and expensive approach, it may not be routinely needed for the small number of mutations that can currently inform thyroid cancer management. [40,41,42]. The major disadvantages of targeting screening are that it requires prior knowledge of the tumor’s genetic makeup and is blind to the acquired mutations that may arise over the course of treatment. However, targeted monitoring can be extremely sensitive, as mutations can be detected at allele frequencies down to 0.01%, with high specificity, in a fast and cost-effective rate manner.

The second strategy used to analyze ctDNA involves high-throughput NGS-based assays to perform untargeted genome-wide screening. Untargeted screening requires no prior knowledge of the cancer’s genomic makeup and identifies novel changes occurring over time. However, it is more costly and time-consuming and requires a relatively large amount of input ctDNA, which is often unattainable. Despite its high sensitivity and specificity, the random PCR error rate using NGS is still 0.1–1% depending on the platform used, which is unacceptably high for the confident identification of rare mutations in ctDNA. Traditional deep sequencing (coverage > 10,000×) can drill down to 0.2% but the requirement for such a high coverage drastically escalates sequencing costs. Duplex sequencing is one of the most accurate methods for detecting low-abundance mutations, achieving 1000-fold fewer errors than standard sequencing, but it is also significantly more costly. By requiring mutations to be present in replicate reads from both strands of each DNA duplex, errors introduced by sample preparation and sequencing can be overcome [43] Another approach, Bias-Corrected Targeted NGS, drastically improves the signal-to-noise ratio by using tags that are connected to each captured DNA fragment, so that every sequencing read is anchored to its clonal family and pull-down probe of origin, facilitating the identification of low-frequency mutant alleles and quantification of subtle changes in gene copy numbers [44].

Mutation-enrichment methods can also be employed to elevate mutation concentrations to levels at which accurate and precise downstream analysis becomes feasible, with less sensitive detection methods. Nuclease-assisted Minor Allele Enrichment with Probe-Overlap (NaME-PrO) [45] and CRISPR-mediated, ultrasensitive detection of target DNA-PCR (CUT-PCR) [46] can be employed to selectively digest WT DNA, and thus alter rare mutations to more readily detectable levels. Both techniques have been demonstrated to enrich point mutations, and NaME-PrO has also proven to be effective for microsatellite instability (MSI) targets [47], while an adaptation called Methylation Specific Nuclease-Assisted Minor-Allele Enrichment (MS-NaME) can target the methylated or unmethylated minority-epigenetic-allele [48].

## 6. Liquid Biopsy in Thyroid Cancer

As in the other cancers described, the emerging role of liquid biopsies in thyroid cancer can provide vital information on somatic and epigenetic mutations, which can guide treatment decision. Early research focused on the gross quantification of cfDNA and detection of CTC. Thyroid cancer patients were found to have longer DNA strands and more cfDNA in their circulation than healthy controls when quantified by qPCR [49].

More recently, liquid biopsy techniques have evolved, and can detect specific mutations, primarily *BRAF^V600E^* and *RET^M918T^* in plasma DNA samples. *BRAF^V600E^*, an oncogenic mutation, is never present in benign disease, yet it is detected in about two-thirds of all differentiated thyroid cancers [50]. Assessment of *BRAF^V600E^* status is now standard in the risk stratification of PTC and an essential determinant of treatment options for anaplastic thyroid cancer (ATC) [51,52,53,54,55]. *RET* rearrangements are the second most common somatic alteration seen in PTC, and occur in up to 40% of PTC [56]. *RET* rearrangements are not seen in normal thyroid epithelial cells [57]. *RET* rearrangement causes the constitutive activation of tyrosine kinase activity through the MAPK pathway. Like *BRAF^V600E^*, *RET* fusions are associated with classic PTC; however, unlike *BRAF^V600E^*, *RET* fusions are not associated with poor prognosis in PTC patients [57]. In contrast to PTC, MTC is associated with single-acid mutations and deletions. MTC occurs sporadically in over 75% of cases, with over 50% harbouring the *RET^M918T^* point mutation. The remaining 25% MTC cases are due to a germline *RET* mutation associated with Multiple Endocrine Neoplasia type 2 [58]. In MTC, knowledge of mutational status is vital for treatment decisions, due to a strong phenotype/genotype correlation.

Liquid biopsies have allowed for these molecular markers to be detected by a simple blood test, opening its role as modality to be used in aiding diagnosis and prognosis, and monitoring treatment response (Table 1).

## 7. Clinical Applications

### 7.1. Mutational Screening

The utility of ctDNA to detect *BRAF^V600E^* mutations has had mixed results, with some studies showing no ctDNA detectioin patients with known *BRAF^V600E^* mutations in both localised and metastatic disease. In a cohort of 22 patients, including four with distant metastasis, who had confirmed *BRAF^V600E^* mutations on tissue samples, no ctDNA was detected using ddPCR or qPCR [59]. In 94 patients, with a mean tumor size of 9.9 mm, the *BRAF^V600E^* mutation could not be detected using qPCR, including four patients with stage 4 disease [60]. More positive studies showed that *BRAF^V600E^ ctDNA* was identified in 42.1% (24/57) patients with PTC and known tumoral mutation using ddPCR, and more often in those with higher risk disease (12/16) [61]. In a study using DNA-mediated clamping PCR, of 49 patients with known *BRAF^V600E^* tumoral mutations, only 6.1% of patients had a concordant ctDNA, all of which had lung metastasis [62]. In more recent years *BRAF^V600E^ ctDNA* was reported to have a 100% concordance in 28 DTC patients with known *BRAF^V600E^* mutation using ddPCR. The variant allele frequency (VAF) of *BRAF^V600E^* in cfDNA ranged from 0 to 2.07% in patients with persistent disease, while it was only 0–0.04% in patients with no evidence of disease. ctDNA levels, when compared to Tg levels, had higher sensitivity and specificity in detecting disease status and tumor burden [63].

The concordance between mutant *RET* ctDNA compared to tissue biopsy has had varied between results. In a cohort of 50 MTC patients who tested positive for *RET^M918T^* following tissue biopsy, the mutation was detected (via ddPCR) in only 32% [64]. Patients with higher levels of mutant *RET^M918T^* in ctDNA, had worse overall survival [64]. In two smaller studies, concordance between ctDNA and tissue was higher, at 61.5–76% [71,72].

In a cohort of 42 advanced thyroid cancer patients with known tumoral mutations, there was a 67% concordance with ctDNA using ddPCR. Concordance rates varied depending on histology, with highest rates in poorly differentiated and anaplastic thyroid cancer (100%). Concordance was 79% for MTC, 60% in follicular thyroid cancer and 53% in PTC.

NGS panels have increasingly been used to detect somatic mutations in thyroid cancer. In 23 patients with ATC, concordance between tissue and ctDNA was the highest in *BRAF^V600E^* (7/10) and *NRAS* (3/4), and in those patients with persistent disease at time of NGS [66]. More recently, the same group compared mutations between cfDNA and tissue in a group of 87 ATC patients on the Guardian 360-73 gene platform [67]. At least one mutation was detected in 92% of patients, with a concordance of 92.9% (26/28) seen for *BRAF^V600E^*, with a PPV of 100%. Patients with cfDNA-detected *PIK3CA* mutation had a worse overall survival than *PIK3CA* WT ATC patients. Notably *TERTp* mutations, present in 65–72.7% of ATC patients [75,76], are difficult to detect in ctDNA, with these mutations found in only 5.8% of 87 ATC patients on NGS testing of cfDNA [67].

### 7.2. Monitoring Treatment Response

The management of thyroid cancer has previously been limited to local therapies, including surgery and radiation therapy. Recently, the development of molecular-based therapies has provided the potential for disease control in patients with advanced and radioactive iodine refractory disease [18,77]. Lenvatinib and sorafenib are multitargeted TKI, and have been shown to improve progression-free survival [17,18]. More specific kinase inhibitors target driver mutations, e.g., selpercatinib, in *RET*-mutated thyroid cancers [18,19,20]. TKIs are associated with significant drug toxicities, including diarrhoea, palmar-plantar-erythroysesthesia and hypertension; therefore, it is vital to determine the optimal time at which to commence therapy, while also closely monitoring the response, to maximise efficacy [78]. ctDNA provides the potential to monitor mutational load while screening for new mutations, which may lead to treatment resistance.

Measuring *BRAF^V600E^* in ctDNA has been found to be effective in monitoring treatment response. In a study of 19 patients, the MAF of *BRAF^V600E^* was significantly reduced 3–6 months following surgery [69]. Similarly, in a cohort of 20 patients with known tumoral *BRAF^V600E^*, seven (35%) had detectable *BRAF^V600E^* on ctDNA at baseline; after three cycles of dabrafenib and trametinib, all seven had undetectable *BRAF^V600E^*, and increased *BRAF^V600E^* ctDNA was then seen in those whose disease subsequently progressed [70].

A study of ctDNA from 34 MTC patients on selpercatinib, which included 13 mutations and 21 fusions, found a 50% reduction in VAF in 79% of sampled patients following treatment, again showing ctDNA’s potential in monitoring treatment response [71]. More recently, eight patients with progressive disease on non-specific *RET* inhibitors, vandetanib or cabozantinib, were found to develop the gatekeeper mutation *RET^V804M^*, which is associated with treatment resistance, using to liquid biopsy [72]. The early identification of resistance mutations is a vital role for the future of ctDNA.

### 7.3. Diagnostic Applications: Assessment of Suspicious Nodules

In large cohort of 103 patients with nodular goitre, ctDNA *BRAF^V600E^* mutations were highest in patients with Bethesda 4 (suspicious of malignancy) and 5 (malignant) nodules [69].

qPCR was used to compare cfDNA tumour strands (67 vs. 180 amplicons) between Bethesda 4 and 5 nodules (*n* = 28) to Bethesda 2 and 3 (*n* = 69) and healthy control (*n* = 49), and found longer tumor stands, associated with higher risk nodules [49].

In contrast, a study of 66 patients with thyroid nodules and no previous history of thyroid cancer found no statistical difference between cfDNA levels in benign and malignant nodules using a 96-mutation NGS panel [73]. In a small study comparing 10 patients with benign nodules to 10 patients with malignant nodules, *BRAF^V600E^* was only detected in patients with malignant nodules on tissues, and not in ctDNA [74].

In 62 patients with early-stage thyroid cancer, *BRAF^V600E^*, *RET*, *NRAS* and *TERTp* mutations were not detected in ctDNA using qPCR; however, one healthy control (1.9%) harboured a KRAS mutation in their plasma sample [68].

## 8. Liquid Biopsies of Epigenetic Markers

Liquid biopsies have evolved in recent years to detect epigenetic markers. Epigenetic makers broadly encompass changes in gene expression that do not result in permanent changes to DNA and include DNA methylation and miRNA. Epigenetic makers often occur early in tumorigenesis and are poised to be future therapeutic targets.

### 8.1. Circulating miRNA

miRNA are short, non-protein-coding RNA transcripts that act to negatively regulate gene expression through messenger RNA (mRNA) translational inhibition or transcript decay [79]. Long, non-coding RNA (lncRNA) transcripts which span more than 200 nucleotides and regulate gene expression by manipulating nuclear architecture, modulating mRNA stability, and regulating mRNA translation and post-translational modification [80]. Aberrant expression of these non-coding RNAs is associated with tumor development in a tissue-specific pattern, which may be exploited to develop diagnostic and prognostic tools. miRNAs have also been proposed as prognostic biomarkers in PTC, with *miR-146b-5p* [81,82,83], *miR-21* [84,85], *miR-1996b-5p* [82], *miR-221* [83,86] and *miR-222* [83,86] overexpression being associated with lymph node and distant metastases. As the lifetime prevalence of lymph node and distant recurrence in PTC patients approximates 20% [87], the miRNA and lncRNA profiling of PTC tumors could be used to tailor patient surveillance of recurrence risk.

The stability of the miRNA shed from tumor cells in bodily fluids and circulating protein complexes makes them attractive as potential non-invasive disease biomarkers [88]. There have been several studies investigating the clinical utility of circulating miRNAs in thyroid cancer (Table 2), in which *miR-451a* [89,90], *miR-146a-5p* [89,91,92] and *miR-222* [93,94] were reproducibly able to differentiate between patients with PTC and benign nodules. Only *miR-222* discriminated between PTC and benign nodules on pre-operative samples (AUC 0.906), with a minimal improvement in diagnostic accuracy using a combined miRNA panel approach (AUC 0.917) [93].

At present, significant obstacles have limited the translation of circulating miRNAs to clinically useful diagnostic tools. Among these, the absence of standardised protocols for sample processing and multiple approaches to the normalisation of expression data have resulted in heterogenous pre-clinical datasets, which are not easily comparable.

### 8.2. Methylation

RAS association domain family protein 1 (RASSF1) and sodium transporter coding gene (SLC5A5) are silenced by promoter methylation in thyroid cancer, with increased methylation levels associated with aggressive disease [99]. In a seminal study, 6689 peripheral blood samples were screened for >1000,000 methylation regions, detecting more than 50 types of malignancy, including thyroid cancer, with a specificity of 99.3% [100]. Methylation signatures were increased in patients with histologically confirmed malignant nodules compared to those with benign pathology [101,102,103].

### 8.3. Future Directions

**Figure 2:**
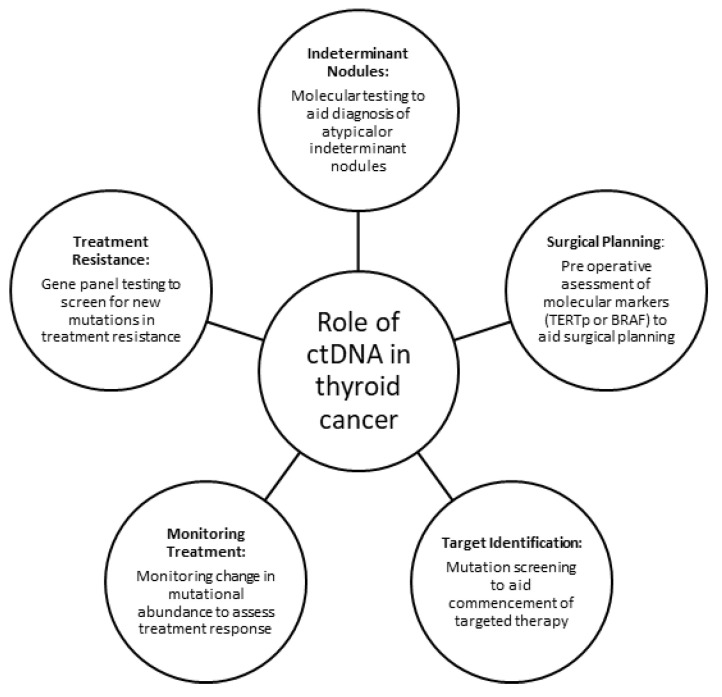
Potential role of liquid biopsy in thyroid cancer.

Liquid biopsy has the potential to become increasingly used for the diagnosis and surveillance of thyroid cancer. As a simple peripheral blood test with the capacity to detect important molecular markers, it has the scope to be used in many clinical applications including aiding in surgical planning, screening for mutations to aid treatment decisions and resistance mechanisms (Figure 2). In the era of targeted therapy, liquid biopsies provide a simple tool and reduce the need for invasive biopsies to gauge eligibility and monitor treatment response. Furthermore, with the increasing incidence of thyroid cancer, cost-effective and low-risk monitoring, and diagnostics with high sensitivity and specificity, will reduce the burden of long-term follow-up. Limitations to its widespread applicability include the low levels of ctDNA in patients with thyroid cancer, and currently limited evidence base, with small sample sizes and variable methods used to detect and report ctDNA. Improved TERTp detection in liquid biopsies, which is notoriously difficult to amplify due to their high GC content, would aid in the prognostication and treatment planning for patients with thyroid cancer. Further studies are also required on the role of other epigenetic markers, including histone modification and copy number alteration, which have been associated with onset and progression in other malignancies and need to be determined for thyroid cancer.

## 9. Conclusions

Liquid biopsy is an emerging technique to screen for molecular biomarkers in peripheral blood samples of cancer patients. Advances in amplification techniques allow for the detection of only a few copies of mutant alleles; however, the inconsistent results and variable methodology between studies have limited its clinical application to date. The potential of liquid biopsies to improve screenings for patients with thyroid cancer warrants ongoing research to improve the technique and allow for its translation from bench to the bedside.

## Figures and Tables

**Table 1:** ctDNA markers for the diagnosis and surveillance of thyroid cancer.

Study	Subjects	Mutation	Detection Technique	Concordance of ctDNA to Tissue	Comments
Mutational Screening					
Condello et al. (2015) [59]	22 PTC (*n* = 4 distant metastasis)	*BRAF^V600E^*	ddPCR qPCR	0%	
Kwak et al. (2013) [60]	94 PTC (*n* = 43; stage 3/4 disease)	*BRAF^V600E^*	qPCR	0%	
Jensen et al. (2020) [61]	57 PTC	*BRAF^V600E^*	ddPCR COLD PCR	42.1%	Tumor size, gross ETE, pulmonary micro-metastases associated with increases
Kim et al. (2015) [62]	49 PTC (*n* = 3 lung metastasis)	*BRAF^V600E^*	PCR	6.1%	100% concordance in 3 patients with lateral LN and lung metastasis
Almubarak et al. (2020) [63]	28 DTC	*BRAF^V600E^*	ddPCR	100%	
Cote et al. (2017) [64]	50 MTC	*RET^M918T^*	ddPCR	32%	Presence of RETm918T associated with worse clinical outcomes and calcitonin doubling in time
Allin et al. (2018) [65]	42 advanced thyroid caner (15 PTC, 14 MTC, 10 FTC, 2 PDTC & 1 ATC)	*BRAF^V600E^* *NRAS* *RET^M918T^*	ddPCR	67% (combined)	
Sandulache et al ( 2017) [66]	23 ATC	*BRAF^V600E^* *NRAS*	NGS	51.4% (combined)	Concordance higher with persistent disease
Qin et al. (2021) [67]	87 ATC	*BRAF^V600E^* *TERTp*	NGS	92.9% (*BRAF^V600E^* naïve) 83.7% (*BRAF^V600E^* previous treatment 5.8% (*TERTp)*	Higher concordance in treatment naive than previously treated
Suh et al. (2021) [68]	62 early stage DTC	*BRAF^V600E^* *TERTp* *NRAS* *KRAS*	qPCR	0% (combined)	
Treatment					
Pupilli et al. (2013) [69]	*N* = 19 patients tested pre and post operatively	*BRAF^V600E^*	qPCR	NA	ctDNA levels reduced 3–6 months post-operatively (*p* < 0.001)
Konda et al. (2017) [70]	7 DTC	*BRAF^V600E^*	ddPCR	NA	Undetectable ctDNA following treatment with dabrafenib
Besse et al. (2018) [71]	29 MTC 9 PTC	*RET*	NGS	76%	Selpercatinib resulted in 50% reduction in variant allele frequency (VAF) in 79% of sampled patients following treatment
Busaidy et al. (2017) [72]	16 MTC	*RET^M918T^*	ddPCR	61.5%	13 patients treated with TKI–8 with progressive disease developed new RET V804M mutations
Suspicious Nodules					
Salvianti et al. (2017) [49]	Bethesda 4 and 5 (*n* = 28) Bethesda 2 and 3 (*n* = 69) Healthy control	cfDNA	qPCR	NA	AUC of 0.765 (*p* < 0.001), 0.982 (*p* < 0.001) and 0.796 (*p* < 0.001) for cfDNA quantity by 67 bp amplicon, cfDNA quantity by 180 bp amplicon and integrity index, respectively
Lupo et al. (2018) [73]	56 nodular diseases (13 malignant, 43 benign)	96 gene Panel	NGS	NA	1/13 malignant and 2/43 benign had positive ctDNA
Pupilli et al. (2013) [69]	103 nodular gotire (*n* = 19 screened with bloods post operatively)	*BRAF^V600E^*	qPCR	NA	AUC = 0.797, *p* < 0.001
Cao et al. (2020) [74]	10 benign nodules 10 malignant nodules	50 amplicon library panel	NGS	0%	

**Table 2:** Circulating miRNA biomarkers for the diagnosis and surveillance of thyroid cancer.

Study	Subjects	Sample	Sampling Time	Normalisation	Analysis Method	Differentially Expressed miRNA		AUC
Yoruker et al., 2016 [95]	31 PTC 31 MNG 24 HC	Serum	Pre-operative 5 weeks post-op	*miR-16* EC	RT-qPCR	↓ in PTC and MNG relative to HC ↓ in PTC postop relative to preop	*miR-21* *miR-31* *miR-151-5p* *miR-221* ***miR-222***	
Zou et al., 2020 [96]	100 PTC 30 MNG 96 HC	Serum exosomes	Pre-operative	C. elegans *miR-39* spike-in control	20 PTC, 20 MNG, 10 HC Screening cohort microarray Training, testing and Validation cohort RT-qPCR	↑ in PTC relative to HC	***miR-25-3p*** *miR-296-5p* *miR-92a-3p* *Combined panel*	0.623 0.621 0.702 0.775
Zhang et al., 2019 [89]	100 PTC 15 MTC 91 BN 89 HC	Serum	Pre-operative	*let-7d/g/I* EC	TLDA screening RT-qPCR validation	↑ in PTC and BN relative to HC ↓ in PTC and BN relative to HC ↑ in MTC relative to BN * and HC	***miR-222-3p*** *miR-17-5p* ***miR-451a*** ***miR-146a-5p*** *miR-132-3p* *miR-183-3p* ***miR-222-3p*** *miR-17-5p* *combined panel*	0.858 * 0.840 * 0.907 *
Graham et al., 2015 [91]	18 PTC 13 BN	Serum	Pre-operative	MS2 bacteriophage RNA spike-in control	RT-qPCR	↓ in PTC relative to BN ↑ in PTC relative to BN	***miR-146a-5p*** *miR-199b-3p* *miR-10a-5p* *let-7b-5p*	
Rosignolo et al., 2017 [92]	44 PTC 19 BN 20 HC	Serum	Pre-operative 30 days post-op	C. elegans *miR-39* spike-in control	11 PTC screening cohort TLDA RT-qPCR validation	↓ in PTC postop relative to preop	***miR-146a-5p*** *miR-221-3p* ***miR-222-3p***	0.653 * 0.730 * 0.587 *
Yu et al., 2012 [93]	106 PTC 95 BN 44 HC	Serum	Pre-operative 5-15 days post-op	*miR-16* EC	Solexa sequencing screening RT-qPCR validation	↑ in PTC relative to BN and HC ↓ in PTC postop relative to preop	*let-7e* ***miR-151-5p*** ***miR-222*** *combined panel*	0.782 * 0.780 * 0.906 * 0.917 *
Ferracin et al., 2015 [97]	27 TC 60 HC	Plasma	Pre-operative	C. elegans *miR-39* spike-in control	ddPCR	↑ in TC relative to HC	*miR-181a-5p*	0.870
Li et al., 2015 [90]	56 PTC 95 BN 10 HC	Plasma	Pre-operative 4-7 days post-op	U6 RNA EC	microarray screening RT-qPCR validation	↑ in PTC relative to BN and HC ↓ in PTC postop relative to preop ^α^	***miR-25-3p**^α^* ***miR-451a**^α^* *miR-140-3p* *let-7i*	0.835 * 0.857 *
Lee et al., 2015 [98]	70 PTC 19 BN	Plasma	Pre-operative	C. elegans *miR-39* spike-in control	RT-qPCR	↑ in PTC relative to BN	*miR-146b* *miR-155*	0.649 * 0.695 *
Kondrotiene et al., 2020 [94]	49 PTC 23 MNG 57 HC	Plasma	Pre-operative 1 month post-op	C. elegans *miR-39-3p* spike-in control	RT-qPCR	↑ in PTC relative to HC ↓ in PTC postop relative to preop	*miR-221* ***miR-222*** *miR-146b* *miR-21* *miR-181b* *miR-21* *miR-221* *miR-146b* *miR-181b*	0.711 *

PTC, papillary thyroid cancer; MNG, multinodular goitre; HC, healthy control; MTC, medullary thyroid cancer; BN, benign nodule; TC, thyroid cancer; EC, endogenous control; TLDA, TaqMan Low-Density Array; ddPCR, digital droplet PCR; ↑, increased expression; ↓ decreased expression. * AUC of PTC relative to BN; ^α^ only these miRNAs had decreased expression postoperatively. miRNAs shown in bold are differentially expressed in multiple studies.

## Data Availability

Not applicable.
